# Association of plasma P-tau181 with memory decline in non-demented adults

**DOI:** 10.1093/braincomms/fcab136

**Published:** 2021-06-14

**Authors:** Joseph Therriault, Andrea L Benedet, Tharick A Pascoal, Firoza Z Lussier, Cecile Tissot, Thomas K Karikari, Nicholas J Ashton, Mira Chamoun, Gleb Bezgin, Sulantha Mathotaarachchi, Serge Gauthier, Paramita Saha-Chaudhuri, Henrik Zetterberg, Kaj Blennow, Pedro Rosa-Neto, for the Alzheimer’s Disease Neuroimaging Initiative

**Affiliations:** 1 Translational Neuroimaging Laboratory, McGill University, Montreal, Canada; 2 Department of Neurology and Neurosurgery, McGill University, Montreal, Canada; 3 Department of Psychiatry and Neurochemistry, University of Gothenburg, Mölndal, Sweden; 4 Clinical Neurochemistry Laboratory, Sahlgrenska University Hospital, Mölndal, Sweden; 5 King’s College London, Institute of Psychiatry, Psychology & Neuroscience, London, UK; 6 NIHR Biomedical Research Centre, London, UK; 7 Department of Epidemiology and Biostatistics, McGill University, Montreal, Canada; 8 Department of Mathematics and Statistics, University of Vermont, Burlington, USA; 9 UK Dementia Research Institute at UCL, London, UK; 10 Department of Neurodegenerative Disease, UCL Institute of Neurology, London, UK

**Keywords:** tau, plasma, Alzheimer’s disease, mild cognitive impairment, memory

## Abstract

Alzheimer’s disease is the leading cause of dementia worldwide and is characterized by a long preclinical phase in which amyloid-β and tau accumulate in the absence of cognitive decline. *In vivo* biomarkers for Alzheimer’s disease are expensive, invasive and inaccessible, yet are critical for accurate disease diagnosis and patient management. Recent ultrasensitive methods to measure plasma phosphorylated tau 181 (p-tau181) display strong correlations with tau positron emission tomography, p-tau181 in CSF, and tau pathology at autopsy. The clinical utility of plasma-based p-tau181 biomarkers is unclear. In a longitudinal multicentre observational study, we assessed 1113 non-demented individuals (509 cognitively unimpaired elderly and 604 individuals with mild cognitive impairment) from the Alzheimer’s Disease Neuroimaging Initiative who underwent neuropsychological assessments and were evaluated for plasma p-tau181. The primary outcome was a memory composite z-score. Mixed-effect models assessed rates of memory decline in relation to baseline plasma p-tau181, and whether plasma p-tau181 significantly predicted memory decline beyond widely available clinical and genetic data (age, sex, years of education, cardiovascular and metabolic conditions, and *APOE*ε*4* status). Participants were followed for a median of 4.1 years. Baseline plasma p-tau181 was associated with lower baseline memory (β estimate: −0.49, standard error: 0.06, *t*-value: −7.97), as well as faster rates of memory decline (β estimate: −0.11, standard error: 0.01, *t*-value: −7.37). Moreover, the inclusion of plasma p-tau181 resulted in improved prediction of memory decline beyond clinical and genetic data (marginal *R*^2^ of 16.7–23%, χ^2^ = 100.81, *P* < 0.00001). Elevated baseline plasma p-tau181 was associated with higher rates of clinical progression to mild cognitive impairment (hazard ratio = 1.82, 95% confidence interval: 1.2–2.8) and from mild cognitive impairment to dementia (hazard ratio = 2.06, 95% confidence interval: 1.55–2.74). Our results suggest that in elderly individuals without dementia at baseline, plasma p-tau181 biomarkers were associated with greater memory decline and rates of clinical progression to dementia. Plasma p-tau181 improved prediction of memory decline above a model with currently available clinical and genetic data. While the clinical importance of this improvement in the prediction of memory decline is unknown, these results highlight the potential of plasma p-tau181 as a cost-effective and scalable Alzheimer’s disease biomarker.

## Teaser

Therriault et al. report that a plasma-based biomarker for phosphorylated tau was associated with memory decline. This biomarker could help predict future cognitive decline associated with Alzheimer’s disease in people **w**ho have mild cognitive symptoms, or no symptoms at all.

## Graphical Abstract

**Graphical Abstract:**
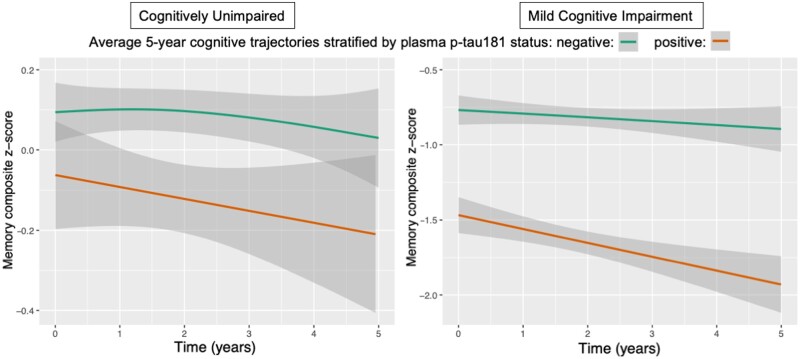

## Introduction

Early and accurate identification of Alzheimer’s disease pathology is critical for clinical trial selection,^1^ disease diagnosis^2^ and increasingly, changes in clinical care.^3^ Currently, *in vivo* quantification of amyloid-β and phosphorylated tau, the core pathological hallmarks of Alzheimer’s disease, is performed with PET or with CSF assays. While these techniques have high sensitivity and specificity for detecting biological Alzheimer’s disease *in vivo*, they are limited by substantial cost, invasiveness and low availability, prohibiting widespread clinical use.

Recent advancements in plasma measurements of phosphorylated tau show high sensitivity and specificity for Alzheimer’s disease pathology, correlate with *in vivo* CSF and PET measures of phosphorylated tau, correlate with tau neurofibrillary tangle burden at autopsy and differentiate Alzheimer’s disease from other dementia syndromes.^4–7^ Biological Alzheimer’s disease is characterized by a preclinical period lasting over a decade in which detectable amyloid-β and tau accumulate in the absence of cognitive symptoms; in familial Alzheimer’s disease, plasma phosphorylated tau concentration starts to increase around 15 years prior to expected clinical disease onset.^8^ Therefore, there exists a promising therapeutic window to identify individuals with biological Alzheimer’s disease for disease-modifying trials. Furthermore, because clinical criteria for Alzheimer’s disease have limited sensitivity and specificity for Alzheimer’s disease pathology,^9^ the addition of plasma-based biomarkers of phosphorylated tau to clinical workups may provide information to physicians evaluating individuals with age-related cognitive impairment.^10^ Plasma assessments of phosphorylated tau may represent a scalable, cost-effective and minimally invasive clinical tool with the potential to improve dementia-related care globally.

Despite the promise of these measurements, large-scale studies of well-characterized cohorts are needed to determine the incremental prognostic information gained from plasma measures of phosphorylated tau.^11^ The goal of this study is therefore to determine associations between plasma measures of tau phosphorylated at threonine181 (p-tau181) with rates of memory decline over up to 5 years in individuals participating in the Alzheimer’s Disease Neuroimaging Initiative (ADNI), a longitudinal multicentre observational study. We also investigated associations between plasma p-tau181 concentration and risk of clinical progression to dementia, in comparison to currently available clinical, cardiovascular and genetic information.

## Materials and methods

### Study design and population

The ADNI is an ongoing prospective cohort study that continues to recruit participants (ClinicalTrials.gov number: NCT01231971); this study included all individuals with available samples for analyses (accessed June 2020). In this study, we assessed cognitively unimpaired (CU; *n* = 509) and amnestic mild cognitive impairment (MCI; *n* = 604) individuals from the ADNI cohort who underwent plasma evaluation of p-tau181 and neuropsychological evaluations. Cognitively normal controls had a Clinical Dementia Rating of 0. MCI subjects had a Clinical Dementia Rating of 0.5, with the memory box score of at least 0.5, with general cognitive performance sufficiently preserved such that a diagnosis of dementia cannot be made by the study physician. The ADNI study was approved by the Institutional Review Boards of all of the participating institutions. Informed written consent was obtained from all participants at each site. Exclusion criteria included neurological diseases other than Alzheimer’s disease, known structural brain abnormalities and history of head trauma followed by persistent neurological symptoms. Full information regarding the ADNI inclusion and exclusion criteria can be accessed at http://adni.loni.usc.edu/ (last accessed June 19th, 2021). Information about access to ADNI data is provided at http://adni.loni.usc.edu/data-samples/access-data/ (last accessed June 19th, 2021).

### Exposures

Clinical predictor variables were age, sex, education and *APOE*ε*4* genotype, as well as a composite cardiovascular and metabolic conditions score. These predictors were selected because of their availability in clinical settings. The cardiovascular and metabolic conditions score was determined as the sum of seven conditions proposed by the US Department of Health and Human Services as indicators of vascular health.^12^ These seven cardiovascular and metabolic conditions were hypertension, hyperlipidaemia, cardiac arrhythmias, coronary artery disease, congestive heart failure, diabetes and stroke,^13^ many of which have been identified as contributors to dementia incidence.^14^^,^^15^ This cardiovascular and metabolic condition score was selected because it has been employed in recent longitudinal population-based studies assessing the relationships between Alzheimer’s disease biomarker status and memory decline^16^ and other studies of ageing and Alzheimer’s disease.^17^ Quantification procedure of plasma p-tau181 concentrations has been described previously.^4^ Assay performance information is provided in the Supplementary material.

### Outcomes

This study’s primary outcome was a numeric memory composite z-score, selected because memory decline is one of the most common concerns with advancing age and an increasingly frequent reason for seeking clinical care in the elderly.^18^ Memory tests consisted of the Wechsler Memory Scale-Revised Logical Memory-II (delayed recall), the Rey Auditory Verbal Learning Task (delayed recall) and the Alzheimer’s Disease Assessment Scale 13-Cog (ADAS-Cog). These tests were selected because they were widely available across ADNI 1, ADNI GO, ADNI 2 and ADNI 3. Each test was converted to z-scores using the mean and standard deviation (SD) of the CU subjects’ baseline evaluation. The composite memory z-score was calculated based on the mean of these three z-scores. Approximately 68% of the CU elderly population had baseline memory composite z-scores within 1 SD of the mean.

### Statistical analyses

Statistical analyses were performed using the R statistical software. Baseline demographic and clinical data were assessed using *t*-tests and χ^2^ tests. Continuous predictor variables were z-scored to reduce the influence of variables with large ranges and to aid in the interpretation of coefficients. In primary analyses, we employed a dichotomized (normal/abnormal) value for plasma p-tau181 because normal/abnormal values are frequently employed in clinical practice to guide patient management^19^ and are often required for clinical trial enrolment.^1^ Individuals were classified into groups of high/low plasma p-tau based on a published threshold of 17.71.^20^ This threshold was obtained from the least distance from (0.1) from a Receiver Operating Characteristic (ROC) curve comparing Amyloid-β negative CU elderly individuals with Amyloid-beta positive Alzheimer’s disease dementia individuals as described in Ref.^20^ Because dichotomizing continuous biomarkers presents with conceptual limitations, we repeated mixed-effect analyses using continuous measures of plasma p-tau181.

We employed mixed-effects models with a continuous memory composite z-score as the outcome with terms for both memory effects at the screening visit, and longitudinal change in memory.^21^ Because of previous reports describing non-linear trajectories of cognitive decline,^22^ we fit quadratic terms for age and time from baseline. The statistical significance of these terms was determined using log-likelihood tests. Time from baseline (years), plasma p-tau181 levels and their interaction were included as fixed effects. Model outputs for plasma p-tau181 provide information about baseline performance and model outputs for the interaction of plasma p-tau181 with time provide information about the longitudinal change (slope). Participants (intercept) and time from baseline visit (slope) were included as random factors.

There were two main steps in statistical modelling in the present study. In the first step, we built a basic clinical prediction model that included readily available clinical information: age, sex, years of education, cardiovascular and metabolic conditions, and *APOE*ε*4* status. While *APOE*ε*4* is not presently recommended for the diagnosis and clinical management of patients with neurodegenerative disease,^14^ it was assessed in order to compare our study with a recent longitudinal study assessing the relationships between Alzheimer’s disease biomarker status and memory decline^16^ which included *APOE*ε*4* status as potentially available clinical information. In this basic clinical model, covariates were evaluated as to whether they were related to memory score at baseline, as well as longitudinal change in memory z-score. In the second step, we built a p-tau181-enhanced model. The p-tau181-enhanced model contained every significant term in the basic clinical model, with the addition of two terms: (i) a dichotomized measurement of baseline plasma-derived p-tau181 to predict baseline memory composite z-score and (ii) interaction between dichotomized baseline plasma-derived p-tau181 with time, to predict longitudinal change in memory z-score. A detailed description of the procedure to build the clinical and p-tau181 models is provided in the Supplementary material.

To compare the basic clinical model with the p-tau181-enhanced model, we compared the marginal *R*^2^ value for each generalized mixed-effect model. Marginal *R*^2^ values correspond to the proportion of the variance that is explained by fixed effects (and does not include variance explained by random effects). We used analysis of variance to compare whether the two models were statistically different. Finally, we report Akaike Information Criterion and Bayesian Information Criterion values for both models.

In exploratory analyses, we added intracranial volume-adjusted hippocampal volume as a neurodegeneration biomarker to the clinical model to determine the incremental information provided by plasma p-tau181 with a more established biomarker. MRI acquisition and processing in ADNI has been described previously (http://adni.loni.usc.edu/methods/mri-tool/mri-pre-processing/, last accessed june 19th 2021). A previously validated adjusted hippocampal volume cut-off of −0.63 cm^3^ was employed to categorize subjects as neurodegeneration positive or negative.^23^

### Survival analyses: Cox proportional hazard models

We employed two separate Cox proportional hazards models to assess time to change in diagnosis from (i) a baseline diagnosis of CU to MCI and (ii) a baseline diagnosis of MCI to dementia. Both models were adjusted for *APOE*ε*4*, age, sex and years of education. All participants were censored at their last follow-up visit. Hazard ratios (HR) are reported. The assumption of proportional hazards was tested using Schoenfeld residuals, non-linearity was assessed using Martingale residuals, and the presence of potentially influential outliers was assessed with Deviance residuals. Survival analyses used binarized values of p-tau181 using the same threshold described above.

### Data availability

Data employed in this study are publicly available and can be accessed through the Laboratory of Neuro Imaging (LONI) database (http://adni.loni.usc.edu/data-samples/access-data/#access_data; last accessed June 19th 2021).

## Results

### Demographics

Demographic and clinical information is summarized in Table 1. Of the 1113 individuals in the study, 535 (48%) were women. The mean (SD) age in the CU group was 73.75 (5.83); the mean (SD) of the MCI group was 72.19 (7.47). Subjects with MCI were more likely to be *APOE*ε*4* carriers. At baseline, individuals with MCI had lower memory composite z-scores than CU individuals. Moreover, individuals with MCI had higher baseline levels of plasma p-tau181. Participants were followed for a median (SD) of 4.1 (1.34) years.

**Table 1:** Demographic and key characteristics of the sample

	CU	MCI	*P*-value
No.	509	604	–
Age, year, mean (SD)	73.75 (5.83)	72.19 (7.47)	<0.0001
Female, no. (%)	260 (51)	257 (42.5)	0.004
Education, year, mean (SD)	16.55 (2.58)	16.03 (2.8)	<0.0001
Racial category			
American Indian/Alaskan Native	1 (0)	1 (0)	
Asian	9 (1.7)	8 (1)	
Black	25 (4.9)	17 (3)	
Hawaiian/Pacific Islander	0 (0)	2 (0)	
More than one	11 (2.2)	6 (1)	
White	460 (90.3)	563 (93)	
Unknown/not reported	2 (0)	7 (1)	
Ethnic category			
Hispanic/Latinx	19 (3.7)	19 (3.2)	
Not Hispanic/Latinx	484 (95)	580 (96)	
Unknown/not reported	4 (0.7)	5 (0.8)	
*APOE ε4 carriers*, %	145 (28.5)	289 (47.8)	<0.0001
MMSE, mean (SD)	29.02 (1.2)	27.25 (3.31)	<0.0001
Baseline memory z-score (SD)	0.00 (0.66)	−1.16 (0.99)	<0.0001
Baseline plasma p-tau181, pg/ml, mean (SD)	16.25 (9.99)	19.01 (12.07)	<0.0001

Mean and standard deviation (SD) are provided for continuous variables and *n* and % are provided for dichotomous variables. *P*-values indicate values assessed with two-sided independent samples *t*-tests for each variable except sex and APOE *ε4* status, where contingency chi-square tests were performed. *P*-values reported are for comparisons to CU subjects.

CU = cognitively unimpaired; MCI = mild cognitive impairment; MMSE = mini-mental state examination; p-tau181 = phosphorylated tau at threonine 181.

### Prediction of memory scores

**Figure 1:**
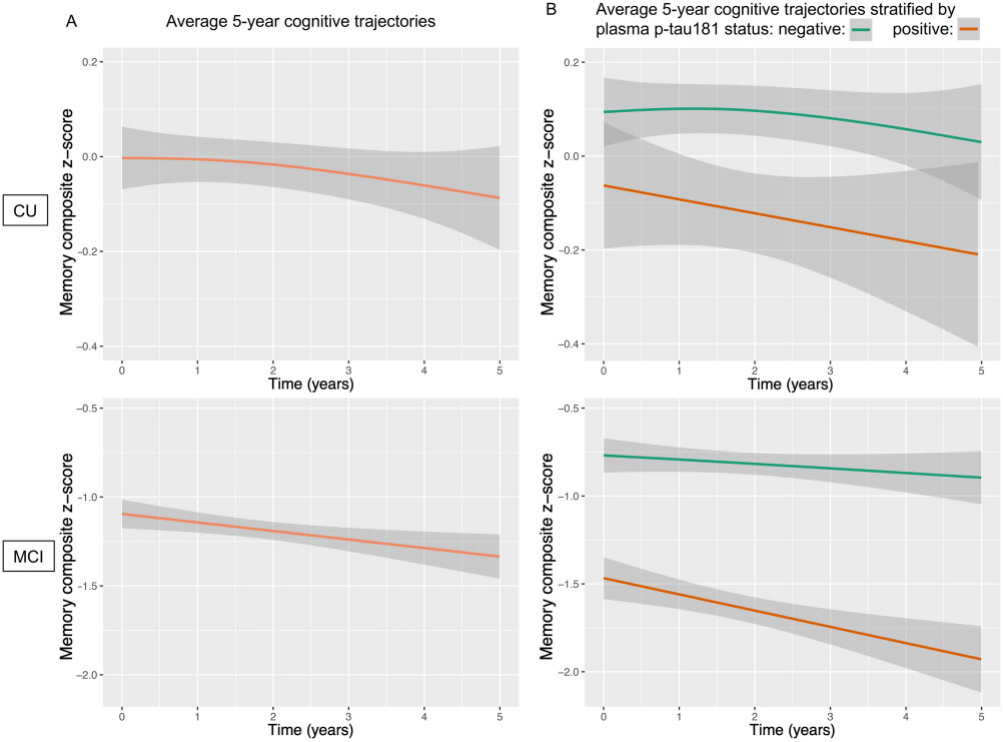
**Association of baseline plasma p-tau181 with 5-year cognitive trajectories. Mixed-effect models predicting memory score at the baseline visit (intercept), as well as longitudinal change in memory score (slope).** (**A**) Average 5-year memory composite z-score decline trajectories for CU individuals (top) and individuals with MCI (bottom). At the group level, CU individuals declined a mean of 0.1 in memory composite z-score, and individuals with MCI declined a mean of 0.2 in memory composite z-scores. (**B**) Five-year memory composite decline trajectories stratified based on baseline plasma p-tau181 status. In CU individuals (top), baseline plasma p-tau181 was not associated with memory performance at baseline (β estimate: −0.1, SE: 0.06, *P* = 0.13), though it predicted faster longitudinal decline over 5 years (β estimate: −0.01, SE: 0.004, *P* = 0.014). In individuals with MCI, elevated plasma p-tau181 was associated with over half a standard deviation lower memory performance at baseline (β estimate: −0.44, SE: 0.07, *P* <0.001), as well as faster rates of decline over 5 years (β estimate: −0.12, SE: 0.02, *P* <0.001) compared to individuals without elevated plasma p-tau181 at baseline.

Plots of group-level memory z-score trajectories over 5 years are presented in Fig. 1A. Figure 1B presents memory z-score trajectories over 5 years grouped by plasma p-tau181 status. In both CU and MCI individuals, positive plasma p-tau181 status was associated with worse baseline memory composite z-scores (β estimate: −0.49, SE: 0.06, *t*-value: −7.97, *P* < 0.001), as well as faster memory decline over 5 years (β estimate: −0.11, SE: 0.01, *t*-value: −7.37, *P* < 0.001). Summary statistics of regression coefficients are summarized in Table 2. Summary statistics for models of CU and MCI groups separately are presented in Supplementary Tables 2 and 3.

**Table 2:** Regression coefficients of p-tau model

	Beta estimate	SE	*t-*value	*P*-value
(Intercept)	−0.07	0.06	−1.32	0.19
Baseline age	1.00	0.47	2.10	0.04
Baseline age^2^	−1.10	0.47	−2.33	0.02
Time (years)	0.09	0.02	4.79	<0.000001
Time (years)^2^	−0.02	0.00	−7.17	<0.000001
Sex, male	−0.30	0.06	−4.94	<0.000001
APOEε4 status, carrier	−0.43	0.06	−7.04	<0.000001
Education, years	0.18	0.03	6.08	<0.000001
Baseline cardiovascular and metabolic conditions	−0.07	0.03	−1.95	0.05
Plasma p-tau181 status	−0.49	0.06	−7.97	<0.000001
Sex × times (years)	−0.01	0.01	−0.38	0.70
Baseline age × time (years)	−0.04	0.01	−5.29	<0.000001
APOEε4 status × time (years)	−0.09	0.01	−6.02	<0.000001
Plasma p-tau181 status × time	−0.11	0.01	−7.37	<0.000001

Summary statistics for p-tau181-enhanced model, in which plasma p-tau181 status is dichotomized based on a threshold of 17.71. Similar results were obtained when using continuous measures of plasma p-tau181 (summarized in Supplementary Table 5).

### Utility of plasma p-tau181 in predicting memory decline

We next compared the incremental utility of adding plasma p-tau181 to the clinical model (consisting of age, sex, education and *APOE*ε*4* genotype, as well as a composite cardiovascular and metabolic conditions score) for predicting memory decline. The p-tau-enhanced model offered significant improvement in the prediction of memory decline over the basic clinical prediction model (χ^2^ = 100.81, likelihood ratio test *P* < 0.001), with marginal *R*^2^ increasing from 16.7% (clinical model) to 23% (p-tau181-enhanced model). Comparative model statistics are presented in Table 3. Elevated plasma p-tau181 was associated with lower memory scores at baseline (β estimate: −0.49, SE: 0.06, *t*-value: −7.97, *P* < 0.001). Furthermore, plasma p-tau181 positivity was associated with faster longitudinal memory decline (β estimate: −0.11, SE: 0.01, *t*-value: −7.37, *P* < 0.001). Non-dichotomized values of plasma p-tau181 yielded a similar improvement in predicting memory decline, with marginal *R*^2^ values increasing to 22.2% in the p-tau181-enhanced model. Comparative model statistics for continuous measures of plasma p-tau181 are presented in Supplementary Table 4, and summaries of regression coefficients when employing continuous measures of plasma p-tau181 are presented in Supplementary Table 5. Subgroup analyses of individuals with subjective cognitive decline are described in the Supplementary material. Analyses comparing the incremental utility of adjusted hippocampal volume are reported in the Supplementary material.

**Table 3:** Comparative model statistics

	No. of parameters	AIC	BIC	Log-likelihood	Deviance	Marginal *R*^2^
Clinical model	16	7779.5	7880.1	−3873.8	7747.5	16.7%
P-tau enhanced model	18	7682.7	7795.9	−3823.3	7645.7	23%

Five-year model comparison for all subjects. Summary model statistics of clinical model and p-tau-enhanced model. The p-tau-enhanced model offered a significantly better prediction of memory decline (χ^2^ = 100.81, df = 2, *P* < 0.00000001).

AIC = Akaike Information Criterion; BIC = Bayesian information criterion.

### Survival analyses

**Figure 2:**
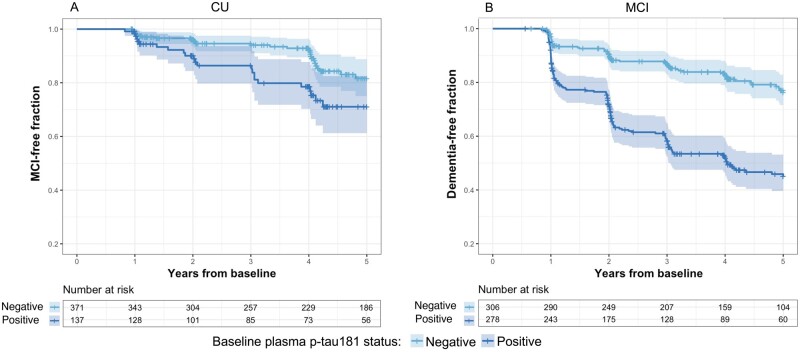
**Association of elevated plasma p-tau181 with clinical progression over 5 years.** (**A)** Kaplan–Meier survival curves for progression from CU to MCI among participants with positive or negative plasma p-tau181 at baseline. Cox proportional hazard models revealed that elevated baseline plasma p-tau181 was associated with an elevated risk of clinical progression to MCI (HR = 1.82, 95% CI: 1.2–2.8, *P*=0.005) in CU elderly individuals. **(B)** Kaplan–Meier survival curves for progression from MCI to dementia among participants with positive or negative plasma p-tau181 at baseline. Cox proportional hazard models revealed that elevated baseline plasma p-tau181 was associated with a higher risk of developing dementia (HR = 2.06, 95% CI: 1.55–2.74, *P* < 0.001) in MCI individuals. Cox proportional hazard models were adjusted for age, sex, years of education, *APOE ε4* status and cardiovascular and metabolic conditions.

We conducted survival analyses to determine associations between plasma p-tau181 status and clinical progression from CU to MCI, or from MCI to dementia. Survival analyses were corrected for age, sex, years of education, cardiovascular and metabolic conditions, and *APOE*ε*4* status. Elevated plasma p-tau181 was associated with increased risk of clinical progression (HR = 2.12, 95% CI: 1.69–2.67, *P* < 0.001) over 5 years in all subjects (Fig. 2). In CU individuals, plasma p-tau181 also predicted progression to MCI over 5 years (HR = 1.82, 95% CI 1.2–2.8, *P* = 0.005). In individuals with MCI, plasma p-tau181 predicted progression to dementia over 5 years (HR = 2.06, 95% CI: 1.55–2.74, *P* < 0.001). Summary statistics for survival models are presented in Supplementary Table 6.

## Discussion

This study presents evidence of associations between plasma biomarkers of p-tau181 and prediction of memory decline over more readily available clinical and genetic information. Higher levels of plasma p-tau181 were related to decreased memory function at baseline. Elevated plasma p-tau181 at baseline also predicted greater memory decline over up to 5 years. A plasma p-tau181 biomarker evaluation provided a significant increase in the prediction of memory decline beyond genetic, clinical and demographic information. Similar results were observed for both continuous and dichotomized values of plasma p-tau181. Taken together, this longitudinal multicentre study provides evidence for the clinical utility of plasma p-tau181 measurements, which are cost-effective, accessible and scalable.

Memory dysfunction is a common reason for seeking medical care in individuals with advancing age.^3^^,^^18^ Plasma p-tau181 levels were associated with a significant improvement in the prediction of cognitive decline over the clinical model consisting of age, sex, education and *APOE*ε*4* genotype, as well as a composite cardiovascular and metabolic conditions score (Marginal *R*^2^ = 16.7% versus 23% by adding plasma p-tau181). This increase in predictive information is comparable to a recent population-based study that reported a 5% increase in the prediction of memory decline over an average of 4.8 years when employing a combination of amyloid-PET, tau-PET and MRI together to classify non-demented individuals based on amyloid-β/tau/neurodegeneration (A/T/N) biomarker status.^16^ While our study was conducted in a highly selected cohort, which may confer optimistic performance for plasma p-tau181, this is a highly encouraging finding given the substantially lower cost and invasiveness of a blood draw as compared to two PET scans and an MRI scan.

Elevated levels of plasma p-tau181 were associated with increased risk of progression from CU to MCI or Alzheimer’s disease (HR = 1.82, 95% CI 1.2–2.8), as well as from MCI to Alzheimer’s disease (HR = 2.06, 95% CI: 1.55–2.74). These HR are somewhat lower than HR reported in CSF studies of clinical progression from CU to MCI or Alzheimer’s disease dementia of 5.21%^24^ or 17.7% for the progression from MCI to Alzheimer’s disease dementia.^25^ Future studies assessing the benefit of the lower invasiveness of plasma p-tau181 in comparison to the higher predictive value conferred by CSF will be needed to further determine the clinical utility of plasma p-tau181.

Plasma p-tau181 is a biomarker specific for Alzheimer’s disease tau pathology and correspondingly does not capture the full spectrum of the A/T/(N) framework as proposed by the National Institute of Aging-Alzheimer’s Association (NIA-AA).^2^ Accordingly, combining plasma assays of p-tau181 with mass spectrometry-based plasma assays of amyloid-β^26^ may result in superior predictions of cognitive and functional decline, especially over longer time periods, as well as superior specificity for Alzheimer’s disease. Indeed, a recent study reported that plasma p-tau181 was only associated with neurodegeneration and cognitive decline in individuals with elevated amyloid-β.^27^ Likewise, the inclusion of a plasma-based neurodegeneration biomarker such as plasma neurofilament light chain,^28^ while not specific for Alzheimer’s disease, may also improve prognostic capacity. In fact, a recent longitudinal study from the BioFINDER and ADNI cohorts reported that a model including plasma neurofilament light chain and p-tau181 was associated with superior prediction of clinical progression from MCI to dementia.^29^ Given recent advancements in blood-based Alzheimer’s disease biomarkers, a single blood sample provides the ability to assess multiple aspects of the Alzheimer’s disease pathological processes, namely amyloid-β, tau and neurodegeneration. This constitutes a promising advantage in contrast to neuroimaging, for which three separate scans would be required to determine a patient’s A/T/N imaging biomarker status,^16^ in turn related to substantially increased cost, patient burden and decreased accessibility.

Early evidence suggests that plasma p-tau181 becomes abnormal approximately 6 years after an individual reaches abnormal levels of amyloid-β,^30^ in line with established biomarker models from autosomal-dominant^31^ and sporadic^32^ Alzheimer’s disease in which amyloid-β abnormality precedes tau abnormality. Recent studies have also associated p-tau181 with cortical thinning as well as hypometabolism,^33^ indicating that plasma p-tau181 may help track neurodegenerative processes in Alzheimer’s disease. However, due to the novelty of plasma-based p-tau measurements, several questions remain.^10^ Studies assessing the positive predictive value of abnormal p-tau181 levels in relation to more established tau biomarkers such as tau-PET^34^^,^^35^ will be required to increase confidence in the significance of plasma p-tau181 concentrations at the individual level.

Our study fits within an emerging body of literature in which plasma concentrations of p-tau181 are associated with cognitive decline. In a recent longitudinal study from the ADNI and BioFINDER cohorts, plasma p-tau181 concentrations predicted clinical progression to dementia from MCI.^29^ Furthermore, a recent longitudinal study also conducted in the ADNI cohort observed associations between plasma p-tau181 and changes in the Preclinical Alzheimer Cognitive Composite in individuals without cognitive impairment.^27^ Taken together, these studies provide evidence for the feasibility of plasma p-tau181 in the individual-level prediction of cognitive decline.

Our study must be interpreted in the context of several limitations. The most important limitation of our study is the selected nature of the ADNI cohort, which constitutes individuals motivated to participate in a study on brain ageing and Alzheimer’s disease and excludes known neurodegenerative diseases other than Alzheimer’s disease. The potential benefit of all diagnostic tests varies with respect to the prevalence of the target condition in the tested population. Plasma p-tau181 measurements are specific for Alzheimer’s disease-type tau^4–6^; therefore, its predictive power is limited to predicting Alzheimer’s disease-related cognitive decline. Because multiple neurodegenerative processes can result in cognitive decline,^36^^,^^37^ plasma p-tau181 will by definition only capture variability in cognitive decline associated with Alzheimer’s disease and will likely be less helpful for predicting cognitive decline in other neurodegenerative diseases. It is therefore important to emphasize that our results cannot be generalized to memory clinic, primary care or population-based cohorts. Future clinical trials in those settings will be needed to determine the utility of plasma p-tau181; the results of our study provide a proof-of-concept that plasma p-tau181 may be useful. However, the specificity of plasma p-tau181 may be beneficial for differential diagnosis, and for screening individuals for disease-modifying trials. Despite the fact that Alzheimer’s disease is the leading cause of dementia worldwide,^14^ investigating the utility of plasma p-tau181 in population-based cohorts and primary care settings is an important next step in determining the utility of this biomarker. Another limitation is the lower proportion of minorities, specifically Black individuals, who participated in this study, a problem reported in other large-scale Alzheimer’s disease biomarkers studies^3^ and pharmaceutical trials.^38–40^ Replicating the present study in a more representative sample is an important step in determining the utility of plasma measures of p-tau181, especially because of recent studies indicating modest relationships between race and tau biomarkers.^41^ It is important to emphasize that as a marker of tau pathology, plasma p-tau181 is not intended to be a complete substitution of the A/T/(N) Alzheimer’s disease biomarker framework, which also includes amyloid-β and neurodegeneration biomarkers. While the goal of this study was to evaluate the additional prognostic value of recently developed plasma measures of p-tau181, we anticipate that with widespread availability of ultrasensitive and specific plasma-derived amyloid-β and neurodegeneration biomarkers, predictive power over clinical and genetic information would likely increase. Moreover, the recent development of plasma measures of tau phosphorylated at other sites such as p-tau217^7^^,^^42^ and p-tau231^43^ deserve investigation. Finally, we wish to emphasize that the thresholds for ‘elevated’ plasma p-tau181 are specific to this study population and future studies are needed to validate appropriate thresholds,^19^ including the possibility of more than one threshold (i.e. low/intermediate/high).

## Supplementary material

Supplementary material is available at *Brain Communications* online.

## Supplementary Material

fcab136_Supplementary_Data

## Abbreviations

ADNI =: Alzheimer’s disease Neuroimaging Initiative
CU =: cognitively unimpaired
HR =: hazard ratios
MCI =: mild cognitive impairment
p-tau181 =: tau phosphorylated at threonine181
