# Supplementary material for: Association of plasma P-tau181 with memory decline in non-demented adults
Source: Brain Commun. 2021 Jun 14;3(3):fcab136. doi: 10.1093/braincomms/fcab136 (PMC8249102; doi:10.1093/braincomms/fcab136)
Supplement: fcab136_Supplementary_Data [file fcab136_supplementary_data.docx]

Supplementary Appendix

Supplement to:

**Utility of plasma p-tau181 to predict memory decline in nondemented adults**

Joseph Therriault BSc^1,2^, Andrea L. Benedet PhD^1,2^, Tharick A. Pascoal MD PhD^1,2^, Firoza Lussier BSc^1,2^, Cecile Tissot BSc^1,2^, Thomas K. Karikari PhD^3,4^, Nicholas J. Ashton PhD^3,4,5,6^, Mira Chamoun PhD^1,2^, Gleb Bezgin PhD^1,2^, Sulantha Mathotaarachchi MSc^1,2^, Serge Gauthier MD FRCPC^2^, Paramita Saha-Chaudhuri PhD^7,8^, Henrik Zetterberg MD PhD^3,4,9,10^, Kaj Blennow MD PhD^3,4^, and Pedro Rosa-Neto MD PhD^1,2^

**Table of contents**

Supplementary methods: Plasma p-tau assay information…………………………………… 2

Supplementary methods: Statistical methods…………………….…………………………… 3

Supplementary methods: Subgroup analyses…………………………………………………. 3

Supplementary results: Terms removed from clinical model…………………………………. 4

Supplementary results: Subgroup analyses……………………………………………………. 4

Supplementary tables………………………………………………………………………….. 6

**Supplementary Methods:**

*Plasma p-tau181*

Plasma p-tau181 was measured using an in-house assay at the Clinical Neurochemistry Laboratory at the University of Gothenburg, Mölndal, Sweden, using an in-house ultrasensitive assay as described previously ^4^. Plasma p-tau181 was measured on Simoa HD-X instruments (Quanterix, Billerica, MA, USA) in April 2020 by scientists blinded to participants’ clinical information. Plasma p-tau181 data was collected over 47 analytical runs. Assay precision was examined by measuring three different quality control samples at the start and end of each run, resulting in within-run coefficients of variation of 3.3%-11.6% and between-run coefficients of variation of 6.4%-12.7%. Out of 3762 ADNI samples, four were excluded from our study due to inadequate volumes. The remaining 3758 all measured above the assay’s lower limit of detection (0.25 pg/ml), with only six below the lower limit of quantification (1.0 pg/ml).

*Statistical methods*

﻿Our statistical modeling procedure is based off of the procedure described in ^12^, which had similar hypotheses as our study, allowing comparisons between studies. Each participant’s “baseline” visit is defined as the first visit with a plasma p-tau181 assessment and memory assessment (response variable). Our analytical framework assessed two aspects: (i) memory composite z-score at the baseline visit (intercept) and (ii) longitudinal rate of change (slope) of memory composite z-score. While longitudinal rate of memory change was the primary outcome measure for the present study, it is desirable to fit both components at the same time ^17^.

Significance of individual model terms were evaluated using likelihood ratio tests. Likelihood ratio test p-values of individual terms were determined by comparing the full model with a nested model that did not contain the term being tested. Because of previous reports identifying quadratic relationships between time and longitudinal cognitive decline ^18^, we also computed a quadratic term for time, as well as every interaction with time. Terms that were not significant are outlined in supplementary table 1 and were removed from the model.

*Subgroup analyses*

We performed a subgroup analysis of CU elderly individuals who reported subjective cognitive impairment (SCI) at baseline (N=339). An individual was classified as having SCI if they met the following criteria: (i) no objective cognitive impairment (CDR of 0; CDR memory box score of 0; MMSE above 24) and (ii) presence of self-reported subjective cognitive impairment as determined by a published cutoff of the Everyday Cognition questionnaire ^21^.

**Results:**

*Terms included and excluded from the clinical model:*

At baseline, men had lower memory scores than women (β estimate: -0.19, SE: 0.06, p=0.003). Cardiovascular and metabolic conditions score was also associated with lower baseline memory scores (β estimate: -0.07, SE: 0.03, p=0.05), as was *APOE* ε*4* carriership (β estimate: -0.53, SE: 0.06, p<0.001). Years of education were associated with higher baseline memory scores (β estimate: 0.21, SE: 0.03, p<0.001). In the clinical model, higher age (likelihood ratio test p<0.001), male sex (likelihood ratio test p=0.0001), and *APOE* ε*4* carriership (likelihood ratio test p<0.001) were associated with faster rates of memory decline. Education (likelihood ratio test p = 0.1) and cardiovascular and metabolic conditions score (likelihood ratio test p = 0.23) were not associated with longitudinal memory decline. Supplementary table 1 presents results of likelihood ratio tests for all terms excluded from the model.

*Subgroup analyses*

When conducting subgroup analyses in CU individuals with subjective cognitive impairment (n=339), the p-tau enhanced model again offered significant improvement in the prediction of memory decline over the basic clinical prediction model (χ^2^ = 7.42, likelihood ratio test p = 0.02), with marginal R^2^ increasing from 17.8% to 19.4%. In CU individuals with subjective cognitive impairment, elevated plasma p-tau181 was not associated with memory scores at baseline (β estimate: -0.06, SE: 0.08, t value: -0.68, p = 0.49). However, elevated plasma p-tau181 was significantly associated with longitudinal memory decline (β estimate: -0.08, SE: 0.03, t value: -2.23, p = 0.02).

*Addition of adjusted hippocampal volume biomarkers*

In CU individuals, abnormal hippocampal volume biomarkers were associated with lower memory scores longitudinally (β estimate: -0.07, SE: 0.03, t value: -2.32, p = 0.02), but not as baseline (β estimate: -0.07, SE: 0.07, t value: -1.055, p = 0.29). In individuals with MCI, abnormal hippocampal volume biomarkers were associated with lower memory scores at baseline (β estimate: -0.31, SE: 0.06, t value: -5.47, p<0.00001) and longitudinally (β estimate: -0.14, SE: 0.03, t value: -5.24, p<0.00001). When comparing adjusted R^2^ values from the clinical model with the MRI-enhanced model in CU, adjusted R2 increased from 12.1 to 13.6% (~1.5% increase). When comparing adjusted R^2^ values from the clinical model with the MRI-enhanced model in MCI, adjusted R^2^ increased from 22.2% to 26.1% (~4% increase). Full model statistics are reported in supplementary tables 7-10.

**Supplementary Tables**

**Supplementary Table 1: P values from Likelihood Ratio tests for terms (covariates) not included in the model:**

| Clinical Model | |
| --- | --- |
| Term | p-value |
| Cardiovascular & metabolic conditions score x time | 0.48 |
| Cardiovascular & metabolic conditions score x time^2^ | 0.23 |
| APOE4 x time^2^ | 0.95 |
| Education, years x time | 0.1 |
| Education, years x time^2^ | 0.72 |
| Age at baseline * x time^2^ | 0.55 |
| Age at baseline^2^ * x time | 0.58 |
| Age at baseline^2^ * x time^2^ | 0.63 |

**Supplementary Table 2: Summary statistics of p-tau181 model in CU**

|  | Beta estimate | SE | *t* value | *p* value |
| --- | --- | --- | --- | --- |
| (Intercept) | 0.25 | 0.05 | 4.728 | <0.001 |
| Baseline age | -0.72 | 0.60 | -1.203 | 0.23 |
| Baseline age^2^ | 0.58 | 0.59 | 0.991 | 0.32 |
| Time (years) | 0.09 | 0.02 | 3.743 | 0.0002 |
| Time (years)^2^ | -0.02 | 0.00 | -4.969 | <0.001 |
| Sex, male | -0.34 | 0.06 | -5.639 | <0.001 |
| APOE﻿ε4 status, carrier | -0.09 | 0.06 | -1.377 | 0.17 |
| Education, years | 0.14 | 0.03 | 4.804 | <0.001 |
| Baseline cardiovascular and metabolic conditions | -0.02 | 0.03 | -0.547 | 0.59 |
| Plasma p-tau181 status | -0.10 | 0.06 | -1.508 | 0.13 |
| Sex x times (years) | 0.01 | 0.02 | 0.48 | 0.63 |
| Baseline age x time (years) | -0.03 | 0.01 | -3.576 | 0.0004 |
| APOE﻿ε4 status x time (years) | -0.04 | 0.02 | -2.414 | 0.02 |
| Plasma p-tau181 status x time^2^ | -0.01 | 0.004 | -2.48 | 0.014 |

Summary statistics for p-tau181 enhanced model in CU elderly individuals only. Plasma p-tau181 status was dichotomized based on a threshold of 17.71.

**Supplementary Table 3: Summary statistics of p-tau181 model in MCI**

|  | Beta estimate | SE | *t* value | *p* value |
| --- | --- | --- | --- | --- |
| (Intercept) | -0.61 | 0.07 | -8.292 | <0.001 |
| Baseline age | -0.40 | 0.54 | -0.734 | 0.46 |
| Baseline age^2^ | 0.21 | 0.55 | 0.391 | 0.69 |
| Time (years) | 0.05 | 0.03 | 2.036 | 0.04 |
| Time (years)^2^ | -0.02 | 0.00 | -4.184 | <0.001 |
| Sex, male | -0.07 | 0.08 | -0.927 | 0.35 |
| APOE﻿ε4 status, carrier | -0.40 | 0.08 | -5.276 | <0.001 |
| Education, years | 0.13 | 0.04 | 3.704 | 0.0002 |
| Baseline cardiovascular and metabolic conditions | -0.08 | 0.04 | -1.875 | 0.06 |
| Plasma p-tau181 status | -0.44 | 0.07 | -5.92 | <0.001 |
| Sex x times (years) | -0.01 | 0.02 | -0.417 | 0.68 |
| Baseline age x time (years) | -0.05 | 0.01 | -4.79 | <0.001 |
| APOE﻿ε4 status x time (years) | -0.10 | 0.02 | -4.714 | <0.001 |
| Plasma p-tau181 status x time | -0.12 | 0.02 | -5.452 | <0.001 |

Summary statistics for p-tau181 enhanced model in individuals with MCI only. Plasma p-tau181 status was dichotomized based on a threshold of 17.71.

**Supplementary Table 4: Comparative model statistics for p-tau181 enhanced model using continuous values of p-tau181**

|  | # of parameters | AIC | BIC | Log Likelihood | Deviance | Marginal R^2^ |
| --- | --- | --- | --- | --- | --- | --- |
| Clinical model | 16 | 7779.5 | 7880.1 | -3873.8 | 7747.5 | 16.7% |
| P-tau enhanced model | 18 | 7695.7 | 7808.9 | -3829.8 | 7659.7 | 22.2% |

Summary model statistics of clinical model and p-tau enhanced model. The p-tau enhanced model offered significantly better prediction of memory decline (χ^2^ = 87.83, df=2, p < 0.001).

**Supplementary Table 5: Summary statistics of continuous p-tau181 model**

|  | Beta estimate | SE | *t* value | *p* value |
| --- | --- | --- | --- | --- |
| (Intercept) | 0.63 | 0.13 | 4.97 | <0.001 |
| Baseline age | 1.16 | 0.48 | 2.43 | 0.02 |
| Baseline age^2^ | -1.25 | 0.47 | -2.64 | 0.008 |
| Time (years) | 0.25 | 0.03 | 7.74 | <0.001 |
| Time (years)^2^ | -0.02 | 0.00 | -7.19 | <0.001 |
| Sex, male | -0.28 | 0.06 | -4.63 | <0.001 |
| APOE﻿ε4 status, carrier | -0.44 | 0.06 | -7.03 | <0.001 |
| Education, years | 0.16 | 0.03 | 5.60 | <0.001 |
| Baseline cardiovascular and metabolic conditions | -0.06 | 0.03 | -1.83 | 0.07 |
| Plasma p-tau181, pg/mL | -0.77 | 0.11 | -7.24 | <0.001 |
| Sex x times (years) | 0.00 | 0.01 | -0.09 | 0.93 |
| Baseline age x time (years) | -0.04 | 0.01 | -5.01 | <0.001 |
| APOE﻿ε4 status x time (years) | -0.09 | 0.02 | -5.74 | <0.001 |
| Plasma p-tau181 pg/mL x time | -0.18 | 0.03 | -7.12 | <0.001 |

Summary statistics for p-tau181 enhanced model using continuous values of plasma p-tau181.

**Supplementary Table 6: Summary statistics of survival models**

|  | HR | CU  95%CI | *p value* |  | HR | MCI  95%CI | *p value* |  | HR | CU + MCI  95% CI | *p value* |
| --- | --- | --- | --- | --- | --- | --- | --- | --- | --- | --- | --- |
| Baseline age | 1.41 | 1.11-1.79 | 0.005 |  | 1.07 | 0.94-1.21 | 0.32 |  | 1.10 | 0.98-1.23 | 0.09 |
| Sex, male | 1.84 | 1.2-2.84 | 0.006 |  | 0.81 | 0.62-1.07 | 0.15 |  | 1.15 | 0.92-1.46 | 0.22 |
| APOE﻿ε4 status, carrier | 1.04 | 0.66-1.63 | 0.87 |  | 1.51 | 1.15-2 | 0.003 |  | 1.46 | 1.16-1.83 | 0.001 |
| Education, years | 0.75 | 0.62-0.92 | 0.005 |  | 1.12 | 0.98-1.27 | 0.1 |  | 0.95 | 0.86-1.06 | 0.36 |
| Cardiovascular/metabolic conditions | 1.03 | 0.8-1.3 | 0.82 |  | 1.08 | 0.93-1.26 | 0.3 |  | 1.05 | 0.93-1.19 | 0.44 |
| Plasma p-tau status | 1.82 | 1.2-2.8 | 0.005 |  | 2.06 | 1.55-2.74 | <0.0001 |  | 2.12 | 1.69-2.67 | <0.0001 |

Summary statistics for p-tau181 enhanced model using continuous values of plasma p-tau181.

**Supplementary Table 7: Summary statistics of MRI model in CU**

|  | Beta estimate | SE | *t* value | *p* value |
| --- | --- | --- | --- | --- |
| (Intercept) | 0.24 | 0.06 | 4.41 | <0.000001 |
| Baseline age | -0.47 | 0.67 | -0.702 | 0.48 |
| Baseline age^2^ | 0.32 | 0.65 | 0.481 | 0.63 |
| Time (years) | 0.10 | 0.03 | 3.052 | 0.002 |
| Time (years)^2^ | -0.02 | 0.01 | -2.277 | 0.02 |
| Sex, male | -0.34 | 0.06 | -5.344 | <0.000001 |
| APOE﻿ε4 status, carrier | -0.11 | 0.07 | -1.64 | 0.10 |
| Education, years | 0.13 | 0.03 | 4.181 | <0.000001 |
| Baseline CMC score | -0.01 | 0.03 | -0.17 | 0.86 |
| Neurodegeneration status | -0.07 | 0.07 | -1.055 | 0.29 |
| Sex x time (years) | 0.02 | 0.03 | 0.898 | 0.37 |
| APOE﻿ε4 status x time (years) | -0.05 | 0.03 | -1.618 | 0.1 |
| Neurodegeneration status x time | -0.07 | 0.03 | -2.325 | 0.02 |

Summary statistics for MRI enhanced model in CU elderly individuals only. Neurodegeneration status was dichotomized according to an adjusted hippocampal volume threshold of -0.63 cm^3^. CMC: cardiovascular and metabolic conditions.

**Supplementary Table 8: Summary statistics of p-tau + MRI model in CU**

|  | Beta estimate | SE | *t* value | *p* value |
| --- | --- | --- | --- | --- |
| (Intercept) | 0.26 | 0.06 | 4.526 | <0.000001 |
| Baseline age | -0.46 | 0.67 | -0.68 | 0.49 |
| Baseline age^2^ | 0.31 | 0.65 | 0.471 | 0.63 |
| Time (years) | 0.10 | 0.03 | 2.982 | 0.003 |
| Time (years)^2^ | -0.02 | 0.01 | -2.022 | 0.04 |
| Sex, male | -0.34 | 0.06 | -5.335 | <0.000001 |
| APOE﻿ε4 status, carrier | -0.11 | 0.07 | -1.54 | 0.12 |
| Education, years | 0.13 | 0.03 | 4.219 | <0.000001 |
| Baseline CMC score | -0.01 | 0.03 | -0.179 | 0.86 |
| Plasma p-tau181 status | -0.07 | 0.07 | -0.963 | 0.33 |
| Neurodegeneration status | -0.07 | 0.07 | -1.048 | 0.29 |
| Sex x time (years) | 0.03 | 0.03 | 0.97 | 0.33 |
| Baseline age x time (years) | -0.05 | 0.03 | -1.562 | 0.11 |
| Plasma p-tau181 status x time | -0.01 | 0.01 | -0.826 | 0.41 |
| Neurodegeneration status x time | -0.07 | 0.03 | -2.135 | 0.03 |

Summary statistics for MRI enhanced model in CU elderly individuals only. Neurodegeneration status was dichotomized according to an adjusted hippocampal volume threshold of -0.63 cm^3^. CMC: cardiovascular and metabolic conditions.

**Supplementary Table 9: Summary statistics of MRI model in MCI**

|  | Beta estimate | SE | *t* value | *p* value |
| --- | --- | --- | --- | --- |
| (Intercept) | -0.72 | 0.07 | -10.405 | <0.000001 |
| Baseline age | -0.25 | 0.52 | -0.476 | 0.63 |
| Baseline age^2^ | 0.10 | 0.53 | 0.186 | 0.85 |
| Time (years) | 0.19 | 0.04 | 5.281 | <0.000001 |
| Time (years)^2^ | -0.03 | 0.01 | -3.528 | 0.0004 |
| Sex, male | -0.02 | 0.07 | -0.345 | 0.73 |
| APOE﻿ε4 status, carrier | -0.33 | 0.07 | -4.735 | <0.000001 |
| Education, years | 0.10 | 0.03 | 2.994 | 0.0029 |
| Baseline CMC score | -0.05 | 0.04 | -1.172 | 0.24 |
| Neurodegeneration p-tau181 status | -0.31 | 0.06 | -5.47 | <0.000001 |
| Sex x times (years) | -0.07 | 0.03 | -2.299 | 0.02 |
| APOE﻿ε4 status x time (years) | -0.17 | 0.03 | -5.856 | <0.000001 |
| Neurodegeneration status x time | -0.14 | 0.03 | -5.24 | <0.000001 |

Neurodegeneration status was dichotomized according to an adjusted hippocampal volume threshold of -0.63 cm^3^. CMC: cardiovascular and metabolic conditions.

**Supplementary Table 10: Summary statistics of p-tau + MRI model in MCI**

|  | Beta estimate | SE | *t* value | *p* value |
| --- | --- | --- | --- | --- |
| (Intercept) | -0.62 | 0.07 | -8.727 | <0.000001 |
| Baseline age | -0.26 | 0.51 | -0.511 | 0.61 |
| Baseline age^2^ | 0.13 | 0.52 | 0.258 | 0.79 |
| Time (years) | 0.23 | 0.04 | 6.286 | <0.000001 |
| Time (years)^2^ | -0.03 | 0.01 | -3.546 | 0.0004 |
| Sex, male | -0.01 | 0.07 | -0.125 | 0.90 |
| APOE﻿ε4 status, carrier | -0.25 | 0.07 | -3.614 | 0.0003 |
| Education, years | 0.11 | 0.03 | 3.138 | 0.002 |
| Baseline CMC score | -0.04 | 0.04 | -1.132 | 0.25 |
| Plasma p-tau181 status | -0.33 | 0.07 | -4.656 | <0.000001 |
| Sex x times (years) | -0.30 | 0.06 | -5.23 | <0.000001 |
| Baseline age x time (years) | -0.06 | 0.03 | -2.039 | <0.000001 |
| APOE﻿ε4 status x time (years) | -0.14 | 0.03 | -4.932 | <0.000001 |
| Plasma p-tau181 status x time | -0.14 | 0.03 | -4.986 | <0.000001 |
| Neurodegeneration status x time | -0.12 | 0.03 | -4.56 | <0.000001 |

Summary statistics for p-tau181 enhanced model in individuals with MCI only. Plasma p-tau181 status was dichotomized based on a threshold of 17.71. Neurodegeneration status was dichotomized according to an adjusted hippocampal volume threshold of -0.63 cm^3^. CMC: cardiovascular and metabolic conditions.
